# Calcium: Alpha-Synuclein Interactions in Alpha-Synucleinopathies

**DOI:** 10.3389/fnins.2016.00570

**Published:** 2016-12-20

**Authors:** Alexandre N. Rcom-H'cheo-Gauthier, Samantha L. Osborne, Adrian C. B. Meedeniya, Dean L. Pountney

**Affiliations:** Menzies Health Institute Queensland, Griffith UniversityGold Coast, QLD, Australia

**Keywords:** Parkinson's disease, α-Synuclein, calcium, Multiple system atrophy, Dementia with Lewy bodies

## Abstract

Aggregation of the pre-synaptic protein, α-synuclein (α-syn), is the key etiological factor in Parkinson's disease (PD) and other alpha-synucleinopathies, such as multiple system atrophy (MSA) and Dementia with Lewy bodies (DLB). Various triggers for pathological α-syn aggregation have been elucidated, including post-translational modifications, oxidative stress, and binding of metal ions, such as calcium. Raised neuronal calcium levels in PD may occur due to mitochondrial dysfunction and/or may relate to calcium channel dysregulation or the reduced expression of the neuronal calcium buffering protein, calbindin-D28k. Recent results on human tissue and a mouse oxidative stress model show that neuronal calbindin-D28k expression excludes α-syn inclusion bodies. Previously, cell culture model studies have shown that transient increases of intracellular free Ca(II), such as by opening of the voltage-gated plasma calcium channels, could induce cytoplasmic aggregates of α-syn. Raised intracellular free calcium and oxidative stress also act cooperatively to promote α-syn aggregation. The association between raised neuronal calcium, α-syn aggregation, oxidative stress, and neurotoxicity is reviewed in the context of neurodegenerative α-syn disease and potential mechanism-based therapies.

## Introduction: α-synuclein in neurodegeneration

α-Synuclein (α-syn) is a small (14 kDa), acidic protein that is highly conserved in vertebrate species. It is primarily expressed in the presynaptic terminals of dopaminergic neurons in the olfactory bulb, frontal cortex, striatum, and the hippocampus. α-Syn has also been observed in hypothalamus, thalamus, midbrain, cerebellum, and the pons/medulla oblongata with lower expression (Maroteaux et al., 1988; Iwai et al., 1995; Clayton and George, 1999). The normal function of α-syn remains unknown, however, it has been found that α-syn can interact with the lipid bilayer of neurons to prevent SNARE-mediated fusion of vesicles (DeWitt and Rhoades, 2013). Cytoplasmic and nuclear inclusion bodies containing α-syn have been found throughout the central nervous system (CNS) in a number of neurodegenerative disorders. These are collectively termed α-synucleinopathies and include Parkinson's disease (PD) and atypical PD, such as multiple system atrophy (MSA) and Dementia with Lewy bodies (DLB; Goedert et al., 2013; Eschbach and Danzer, 2014; Radford et al., 2014). Aggregation of α-syn results from dynamic instability of the native structure and may be promoted by gene mutations or by environmental stressors, such as oxidative stress or metal ion concentration changes.

**Figure 1:**
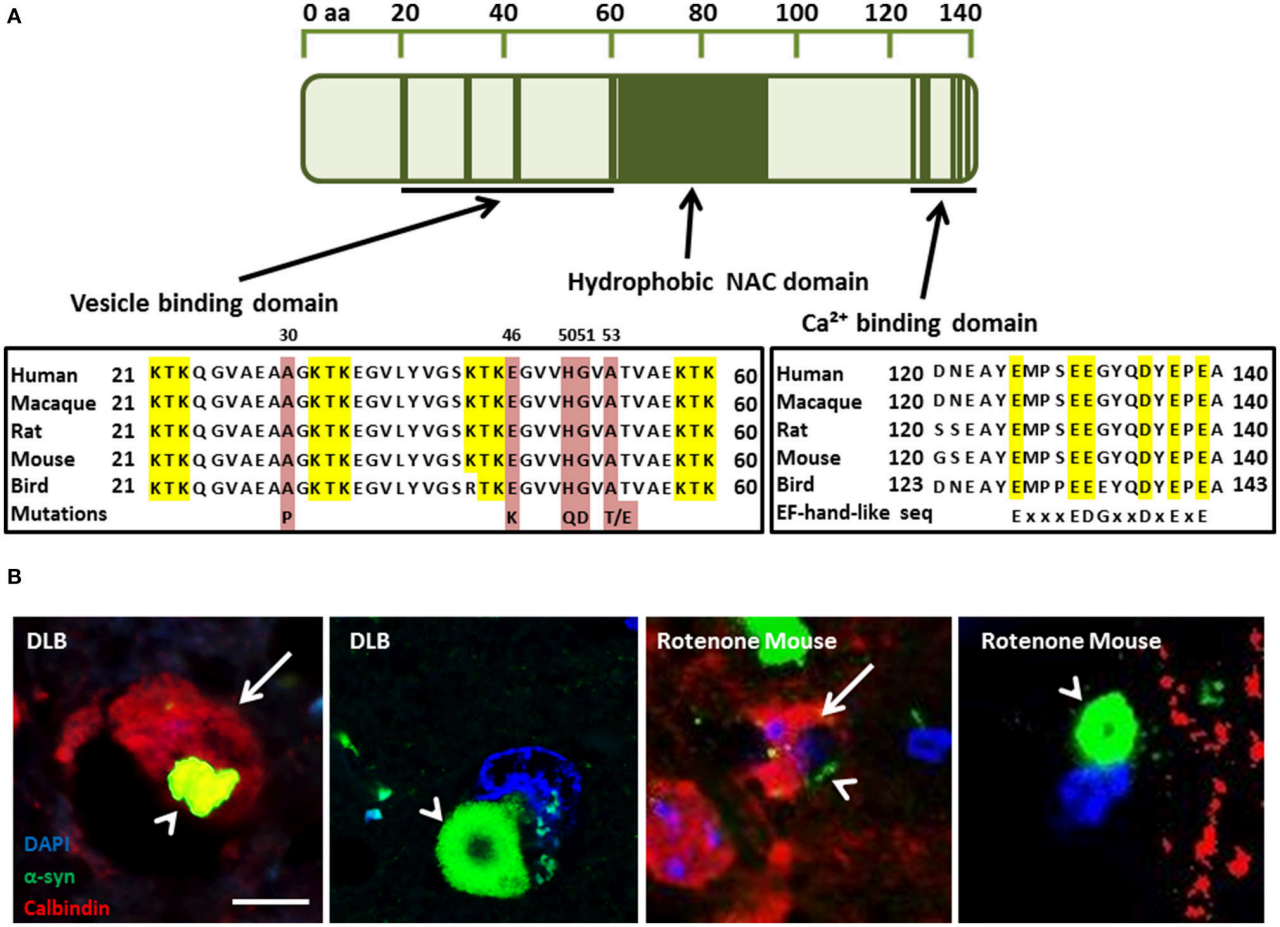
**(A)** α-Synuclein (α-syn) protein domain structure. KTK repeats in the N-terminus are involved in lipid interaction, the hydrophobic NAC domain is important for aggregation and the C-terminal Ca(II) binding site can increase the rate of oligomerization (Nielsen et al., 2001; Lowe et al., 2004). Parkinson's disease linked point mutations are indicated within the neurotransmitter vesicle binding domain. **(B)** Neuronal Calbindin-D28K (CB), inhibits α-syn inclusion bodies in DLB and mouse model tissue (Rcom-H'cheo-Gauthier et al., 2016). (B1-2) Dementia with Lewy bodies immunofluorescence for α-syn (green) and CB (red, arrow) showing rare, small cytoplasmic α-syn aggregates (arrowhead) in a CB+ neuron and a typical, large perinuclear α-syn inclusion body in a CB- neuron. (B3-4) Rotenone lesioned mouse (oxidative stress) model shows a similar pattern of large perinuclear α-syn inclusion bodies that are absent from CB expressing neurons, with only small α-syn puncta rarely detected in CB positive cells; scale bar 5 μm.

PD can be classified into two main groups: idiopathic PD, accounting for 85–90% of all PD cases; and familial PD, accounting for 10–15%. Currently, six PD-linked mutations in PARK1/4, the α-syn gene, have been identified that may provide important clues to the pathways that may give rise to idiopathic PD. They include the A30P (Kruger et al., 1998), A53T (Polymeropoulos et al., 1997), E46K (Zarranz et al., 2004), H50Q (Proukakis et al., 2013), A53E (Pasanen et al., 2014), and G51D (Lesage et al., 2013) amino acid substitutions that disrupt the membrane binding domain (Figure 1A). The A53T and A30P mutations also alter neuronal cytotoxicity in response to hydrogen peroxide and 1-methyl-4-phenylpyridinium (MPP+) treatment. Expression of these mutant isoforms significantly increased cytotoxicity in comparison to cells expressing wild-type (WT) α-syn, which was similar to control cells (Kanda et al., 2000). Furthermore, both mutations showed increased fibril formation compared to WT (Conway et al., 1998, 2000; Narhi et al., 1999). E57K: mThy-1 human α-syn transgenic mice with increased oligomerisation of α-syn had decreased vesicles, synapse, and neuron degeneration in comparison to wildtype controls and E57K mice expressing lower levels of α-syn (Rockenstein et al., 2014). Gene duplication (Chartier-Harlin et al., 2004; Ibanez et al., 2004) and triplication of α-syn (Singleton et al., 2003) has also been found in familial PD, implicating increased expression of the wild-type protein. Recasens et al. (2014) have demonstrated that α-syn species found in PD-derived Lewy bodies (LBs) were pathogenic and had the capacity to initiate a PD-like pathological process. This included intracellular and presynaptic aggregation of pathological α-syn species in various parts of the brain and progressive cell loss of dopaminergic neurons. Several metals have been implicated in the aggregation of α-syn, including, iron, manganese, copper, and zinc (Santner and Uversky, 2010; McAllum and Finkelstein, 2016). However, this review will focus on calcium in alpha-synucleinopathies and its interaction with α-syn.

## Voltage-gated Ca(II) channels in neurodegeneration

Homeostatic Ca(II) regulation is essential to the health and functioning of cells. Although N-type voltage-gated Ca(II) channels (Ca_v_) are the primary regulators of Ca(II) influx and corresponding neurotransmitter release in neurons (Simms and Zamponi, 2014), various studies highlight the broader range of Ca(II) channels found throughout the CNS. There are three types of Ca_v_ expressed in the brain, Ca_v_1, Ca_v_2, and Ca_v_3 (Catterall et al., 2005), comprising four Ca_v_1 subtypes (L-type configuration), three types of Ca_v_2 channels (P/Q, N, and R configuration), with Ca_v_2.1 and Ca_v_2.2 responsible for neurotransmitter release, and the family of low-voltage activated T-type channels, Ca_v_3.1, Ca_v_3.2, and Ca_v_3.3 (Talley et al., 1999; Heady et al., 2001; Catterall et al., 2005). Inhibition of Ca_*v*_1, 2.1, and 2.2 channels have each been shown to decrease neuronal damage upon brain injury (Perez-Pinzon et al., 1997; Babu and Ramanathan, 2011; Kim et al., 2016), which also leads to increased α-syn expression, α-syn aggregation, and Parkinson's-like pathology (Acosta et al., 2015). Ca_*v*_3 contribute to the pathogenesis of epilepsy (Chemin et al., 2001), and have also been reported in glial cells (Yunker et al., 2003). Bassoon, a neuronal zinc finger pre-synaptic scaffold protein shows increased expression in MSA cerebellum (Hashida et al., 1998). Interestingly, Bassoon increased Ca_v_2.1 expression, associated with a longer calcium transient, when over-expressed and decreased Ca_v_2.1 when knocked out in an epilepsy model (Davydova et al., 2014; Ivanova et al., 2015). Moreover, pathological α-syn species may mediate calcium entry and thus both further promote calcium-dependent α-syn aggregation and directly lead to calcium-dependent cytotoxicity (Angelova and Abramov, 2016). Wildburger et al. (2009) investigated the neuroprotective properties of the Ca(II) channel blocker, trimethadione (TMO), in cultured hippocampal and cortical neurons from C57BL/6 and α1H^−/−^ mice, showing a significant decrease in cell death following TMO treatment (Wildburger et al., 2009). Moreover, Follett et al. (2013) showed that SH-SY5Y cells subjected to membrane depolarization by potassium resulted in Ca(II) influx and α-syn-positive cytoplasmic aggregates that could be blocked by TMO pre-treatment. Furthermore, Danish subjects taking dihydropyridine L-type calcium channel blockers between 1995 and 2006 were 27% less likely to develop PD (Ritz et al., 2010; Pasternak et al., 2012). Following this, a Phase II tolerability trial in early stage PD patients was conducted and found that isradipine was well-tolerated (Parkinson Study Group, 2013).

## Ca(II) regulation in ageing and neurodegeneration

Regulation of calcium homeostasis requires a complex interplay between the cytoplasmic channels/pumps and intracellular Ca(II) stores, such as the mitochondria and endoplasmic reticulum (ER), and may be affected by age (Toescu and Verkhratsky, 2007). It has been established that resting intracellular Ca(II) levels remain the same despite the age of the neuron. However, the return time to resting levels is significantly increased in aged neurons after a stimulus. Aged neurons also exhibit a decreased capacity to recover from Ca(II) stimulus through uptake into the intracellular Ca(II) stores with a decline in sarco/endoplasmic reticulum Ca(II)-ATPase (SERCA) function (Pottorf et al., 2000). Moreover, Ca(II) pumping into the extracellular space via the plasma membrane Ca(II) ATPase (PMCA) has been shown to be impaired in aged neurons (Michaelis et al., 1996). The calcium buffering capacity of neurons was also shown to be decreased in aged neurons and some studies have shown that after stimulation, the concentration of Ca(II) may be decreased, but the recovery time back to resting levels is dramatically increased (Duckles et al., 1996). Calbindin D28K (CB), calretinin (CR), and parvalbumin (PV) are three cytosolic Ca(II) buffering proteins (CBP) that are capable of Ca(II) buffering in neurons and belong to the EF-hand protein family. The other CBP family is the annexin family and is characterized by proteins that bind Ca(II) in the presence of phospholipids-containing membranes (Andressen et al., 1993). The EF-hand proteins may function either as Ca(II) buffers, decreasing the free cytoplasmic concentration, or as triggers starting a cascade of responses (Dalgarno et al., 1984). The ubiquitous trigger protein calmodulin activates at least 20 different enzymes, while PV, CB, and CR, are more passive buffers decreasing the amplitude of Ca(II) signals. In a human study conducted by Bu et al. (2003), a decrease was found in both CR and CB in aged compared to young cortical neurons, but no difference in PV positive neurons. Yamada et al. (1990) found that dopaminergic neurons of the Substantia nigra pars compacta (SnPc) that were high in CB were preferentially spared in control brain sections compared with PD patients. The importance of these proteins has also been implicated by German et al. (1992) who studied the brains of human patients with PD, MPTP monkey or in C57BL/6. They found that in both idiopathic PD and in the above models, neurons that contained CB were spared while neurons in CB-negative (CB-) regions were lost. Gaspar et al. (1994) found that dopaminergic neurons expressing CB were spared from loss in a mouse model with Parkinsonian-like pathological features. Tsuboi et al. (2000) and Kim et al. (2000) found that CR expression in dopaminergic neurons of the SnPc were more protected against 6-hydroxydopamine (Kim et al., 2000; Tsuboi et al., 2000). Moreover, a recent study has described the over-representation of CB-positive (CB+) neurons in DLB, and the almost complete exclusion of α-syn aggregates in CB+ neurons. This study draws the same conclusions in a rotenone (oxidative stress) mouse model of α-synucleopathy (Rcom-H'cheo-Gauthier et al., 2016). In both DLB and the mouse model, occasional neurons with low CB expression showed small α-syn aggregates, but large perinuclear inclusion bodies were only detected in CB- cells (Figure 1B; Rcom-H'cheo-Gauthier et al., 2016).

## Increased intracellular Ca(II) induces α-synuclein oligomerisation

Nielsen et al. (2001) first demonstrated that Ca(II) binding to α-syn regulates ligand binding and oligomerization. Subsequently, Lowe et al. (2004) found that calcium promoted α-syn annular oligomer formation that required the C-terminal calcium binding site (Figure 1A). The α-syn C terminus contains a sequence of six negatively charged acidic amino acids similar to an EF-hand-like motif (Zhou et al., 2006), although more studies are required to precisely delineate the calcium binding residues. More recent studies have shown that by increasing free intracellular Ca(II) concentration thapsigargin or Ca(II) ionophore chemical treatments caused a significant increase in the proportion of cells bearing microscopically-visible α-syn aggregates. It was also demonstrated that co-treatment with intracellular Ca(II) chelator, suppressed the aggregation, indicating that α-syn aggregation was induced by raised intracellular free Ca(II) directly, although a possible role for calpain protease activation could not be excluded. Moreover, supporting studies with recombinant α-syn indicated that direct binding of Ca(II) ion to α-syn promoted rapid oligomer formation *in vitro* (Nath et al., 2011). More recently, Follett et al. (2013) demonstrated that depolarization of the plasma membrane resulted in raised intracellular free Ca(II) and α-syn aggregation triggering the formation of large LB-like perinuclear α-syn inclusion bodies after 48 h that could be inhibited both by Ca(II) chelation and calcium channel blockade (Follett et al., 2013). Moreover, Chan et al. (2007) assessing Ca(II) blockade using brain slices from a MPTP mouse model of PD determined that the L-type Ca_*v*_1.3 Ca(II) channel inhibitor, Isradipine, a drug used to treat high blood pressure, could recover dopaminergic neural activity. Furthermore, Singh et al. (2016) also used an L-type calcium channel blocker, nimodipine, in the same model and observed reduced loss of TH positive neurons and reduced reactive oxygen species (ROS) production in the striatal mitochondria.

## Oxidative stress and α-synuclein oligomerization

Another potential target for α-synucleinopathy therapeutics could be to target oxidative stress in the brain. There is evidence that oxidative stress is increased in the normal aged brain, however the level of oxidative stress is greatly increased in patients with neurodegenerative diseases (Sims-Robinson et al., 2013). Thus, WT α-syn has been shown to induce mitochondrial NO when it is associated with mitochondria (Parihar et al., 2008). This indicates that not only will normal increases in oxidative stress cause aggregation but that aggregation of α-syn also induces more oxidative stress within the cell forming a positive feedback loop. Moreover, other research shows that α-syn protects cells from oxidative stress by deactivating the c-jun N-terminal kinase (JNK) pathway, although this data was from cells challenged with exogenous H_2_O_2_ (Hashimoto et al., 2002). The combination of oxidative stress and α-syn expression has been used to generate a model of MSA in mice, whereby the over-expression of α-syn in glial cells is combined with 3-nitropropionic acid. Treatment to induce mitochondrial oxidative stress was sufficient to induce MSA like pathology (Ubhi et al., 2009). Moreover, Kume et al. (2012) found urinary 8-OHdG levels were significantly higher in DLB cases compared to controls, indicating systemically increased oxidative stress. Oxidative stress in aging primates originates primarily from mitochondrial complexes I and III of the electron transport chain which leads to greater mitochondrial DNA damage compared to nuclear DNA (Castro Mdel et al., 2012). Indeed, widespread mitochondrial DNA damage occurs at early stages of DLB (Lin et al., 2012). Moreover, α-syn oligomers promoted Ca(II)-induced mitochondrial dysfunction (Luth et al., 2014) and oxidative stress characterized by α-syn lipoxidation precedes the formation of α-syn aggregates and the development of neocortical LB pathology in DLB (Dalfo and Ferrer, 2008). Furthermore, Quilty et al. (2006) showed that when mouse primary neocortical cells were incubated in the absence of antioxidants, mild oxidative stress caused α-syn accumulation in a subset of neurons. Indeed, recent studies have found the oxidized form of the endogenous oxidative stress sensor, DJ-1, progressively increased in the later stages of PD and more highly oxidized forms were likely present in DLB (Saito et al., 2014). Furthermore, it has been demonstrated that Ca(II) influx can interact with α-syn to mediate increased oxidative stress (Dryanovski et al., 2013; Surmeier et al., 2016). Indeed, the combination of oxidative stress and raised intracellular free Ca(II) showed a synergistic effect on α-syn aggregation (Goodwin et al., 2013). More recently, α-syn aggregation was studied using an oxidative stress mouse model where unilateral lesion was performed using the mitochondrial complex I inhibitor, rotenone. The results showed an increase in α-syn aggregates in the lesioned hemisphere, compared to sham or unlesioned, especially in aged animals, combined with an exclusion of α-syn aggregates from CB-expressing neurons, further implicating a cooperative interaction between calcium buffering and oxidative stress (Rcom-H'cheo-Gauthier et al., 2016).

## α-synuclein aggregation, raised Ca(II), and oxidative stress

Hettiarachchi et al. (2009) demonstrated that elevated levels of intracellular α-syn elevated levels of intracellular Ca(II), although reduced Ca(II) was observed in primary neurons overexpressing α-syn when treated with manganese (Dučić et al., 2015). Furthermore, secreted α-syn induced an increase in capacitive Ca(II) entry in differentiated SH-SY5Y cells (Melachroinou et al., 2013). Transmembrane entry of Ca(II) or other cations may be via α-syn pore-like oligomers (Pountney et al., 2005; Mironov, 2015) and can mediate cytotoxicity (Angelova et al., 2016). Transgenic mice over-expressing human WT α-syn showed that α-syn transgenic mice exhibited augmented, long-lasting Ca(II) transients characterized by considerable deviation from the exponential decay. Furthermore control and α-syn knock out groups demonstrated low percentages of neurons with Ca(II) abnormalities whereas the α-syn transgenic group showed Ca(II) response alteration, suggesting these alterations are related to α-syn expression (Reznichenko et al., 2012; Mironov, 2015). Furthermore, Navarria et al. (2015) showed that overexpression of α-syn reduced NMDAR-mediated Ca(II) influx.

Cali et al. (2012) observed significantly greater levels of mitochondrial Ca(II) in α-syn treated cells compared to control cells. In SH-SY5Y and HeLa cells, ER-mitochondria interactions and increased mitochondrial Ca(II) were enhanced by overexpressed α-syn and increased mitochondrial Ca(II) transient. Moreover, treatment with native α-syn increased Ca(II) entry in primary rat cortical neurons and induced mitochondrial Ca(II) uptake. Dryanovski et al. (2013) found that in dopaminergic neurons bearing inclusions, ROS levels in the mitochondria were greater in the soma and proximal dendrites than in neurons not bearing any inclusions. Treatment with isradipine significantly reduced the ROS levels in CB− dopaminergic neurons when compared to CB+ dopaminergic neurons, suggesting ROS production in the cytosol is triggered by the formation of α-syn inclusions. Buttner et al. (2013) established that α-syn leads to elevated cytosolic Ca(II) in yeast that coincides with an increase in oxidative stress. This suggests that the formation of α-syn aggregates triggers an increase in mitochondrial Ca(II) transient and then leads to oxidative stress. WT α-syn also displayed the capacity to induce mitochondrial NO when associated with mitochondria (Parihar et al., 2008) and oxidative stress induced by Ca(II) influx was worsened in a DJ-1 mutant mouse PD model (Goldberg et al., 2012). Thus, an increase in oxidative stress can cause α-syn aggregation and that aggregation can also induce further oxidative stress within the cell forming a positive feedback loop. Though, this contradicts other research by Hashimoto et al. (2002) which shows that α-syn protects cells from oxidative stress by inactivating the JNK pathway. More recently, the antioxidant capacity of SH-SY5Y cells was shown to be decreased by changes in SOD1 and SOD2 enzyme activity and decreased gluthathione levels following overexpression of α-syn (Perfeito et al., 2016). Koch et al. (2016) also found that α-syn fibrils increase SOD1 aggregation *in vitro* and *in vivo*.

**Figure 2:**
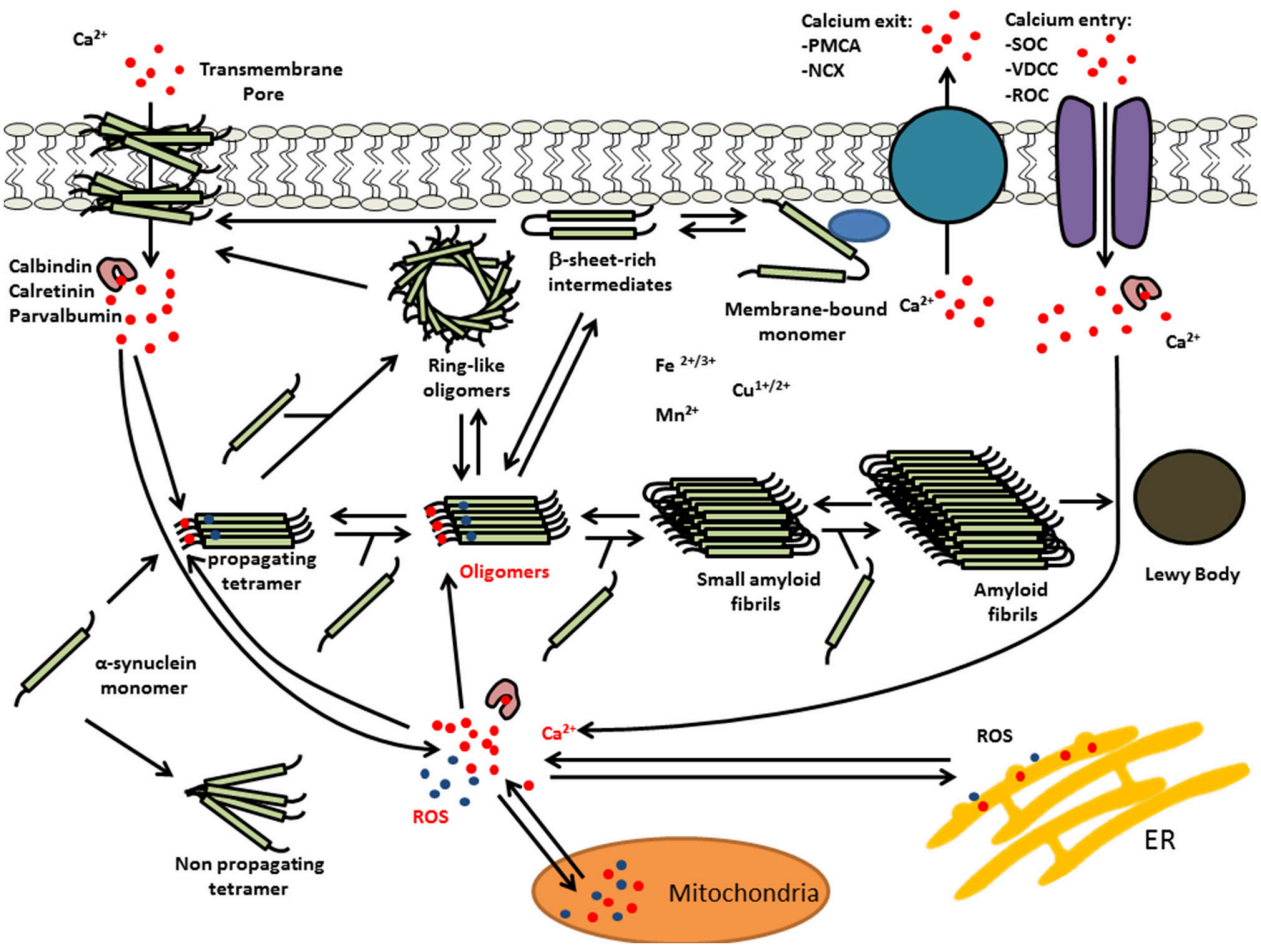
**Multiple interactions of Ca(II) and oxidative stress on α-syn aggregation**. Elevated levels of intracellular Ca(II) may cause α-syn aggregation and the formation of LBs. This calcium dysregulation could be mediated by elevated reactive oxygen species (ROS) and/or calcium mobilization from mitochondria, α-syn oligomer pores or dysregulation of voltage-dependent calcium channel (VDCC), plasma membrane Ca(II) ATPase (PMCA), or Na(I)-Ca(II) exchanger (NCX).

Recent findings indicate that oxidative stress and increased intracellular free Ca(II) synergistically increased the number of α-syn-positive aggregates (Goodwin et al., 2013). Moreover, *in vitro* experiments demonstrated that Ca(II) binding alone was able to induce non-stable α-syn aggregates, whereas, co-treatments with Ca(II) and oxidant resulted in the formation of larger, stable aggregates. Furthermore, kinetic experiments of α-syn aggregate formation by Nath et al. (2010) were consistent with an auto-catalytic mechanism. Thus, Ca(II)/oxidation stabilized α-syn aggregates could in turn serve to increase nucleation centers in disease. This is supported by work performed by Krishnan et al. (2003) that show that oxidatively di-tyrosine cross linked α-syn dimers were the rate limiting step in the fibrillation process in forming nucleation centers. Moreover, Ca(II) influx into neurons can induce oxidative stress that may in turn result in further α-syn aggregation. Figure 2 summarizes the potential synergies between raised calcium levels, oxidative stress, and α-syn aggregation.

## Conclusion: challenges for future therapeutics

Calcium channel antagonists are currently undergoing clinical trials for PD therapy, however many of these drugs may display undesirable features when used chronically. Modulating neuronal calcium homoeostasis via endogenous CBP or through differential calcium channel recruitment could offer efficient alternatives.

## Author contributions

DP: Research group leader coordinated writing, editing of text and figures. AR: lead author contributed majority of text. SO: contributed some text. AM: reviewing co-author.

### Conflict of interest statement

The authors declare that the research was conducted in the absence of any commercial or financial relationships that could be construed as a potential conflict of interest.

## Abbreviations

α-syn: α-synuclein
CB: Calbindin D28K
CBP: Calcium buffering protein
CNS: Central nervous system
CR: Calretinin
DLB: Dementia with Lewy bodies
ER: Endoplasmic reticulum
GC: Glial cytoplasmic inclusion
JNK: c-jun N-terminal kinase
LBs: Lewy bodies
MSA: Multiple system atrophy
NCX: Na(I)-Ca(II) exchanger
PD: Parkinson's disease
PMCA: Plasma membrane Ca(II) ATPase
PV: Parvalbumin
SNpc: Substantia nigra pars compacta
ROS: Reactive oxygen species
TMO: Trimethadione
VDCC: Voltage-dependent calcium channel
SERCA: sarco/endoplasmic reticulum Ca^2+^-ATPase.
